# AI performance varies considerably across mammography devices: a multi-site and multi-vendor retrospective study

**DOI:** 10.1186/s13244-026-02383-5

**Published:** 2026-08-26

**Authors:** Marcel Blum, Rudolf Morant, Alena Eichenberger, Alexander Geissler, Jonas Subelack, Justus Vogel, David Ehlig

**Affiliations:** 1 Chair of Health Economics, Policy and Management, School of Medicine, University of St.Gallen, St.Gallen, Switzerland; 2 Cancer League of Eastern Switzerland, St.Gallen, Switzerland

**Keywords:** Artificial intelligence, Breast neoplasms, Equipment design, Mammography, Sensitivity and specificity

## Abstract

**Objectives:**

To evaluate an artificial intelligence (AI) algorithm on three different mammography devices and assess screening performance using general and device-specific thresholds.

**Materials and methods:**

This retrospective study analyzed 22,673 AI-assessed mammographies of women aged 50–69 collected in 2022–2023 from three mammography devices across six screening sites within a Swiss Mammography Screening Program. 115 screen-detected and 15 1-year interval breast cancers were included in the analyses. A commercially available AI algorithm assessed each mammography with a numeric case score. Optimal thresholds were determined across all devices and for each device separately using the Youden-Index. Screening performance was assessed using sensitivity, specificity, balanced accuracy, and cancer detection rate (CDR).

**Results:**

Standard double-reading had a CDR of 5.07 (95% CI: 4.19–6.09). Using AI with a general threshold achieved a CDR of 4.81 (95% CI: 3.95–5.80), which was enhanced using device-specific thresholds to 4.90 (95% CI: 4.03–5.89). Workload for radiologists in consensus conferences was lowered by nearly one-third when using device-specific thresholds compared to a general threshold (*p* < 0.001). Nonetheless, AI screening performance for one of the mammography devices was poor even with device-specific thresholds.

**Conclusion:**

AI-based performance varied considerably among the three mammography devices, emphasizing the need for device-specific thresholds or even the withdrawal of AI use on certain devices. Awareness of different case distribution among radiologists should be raised to mitigate the risk of interpretation biases and reduced diagnostic accuracy. Lastly, training datasets must be representative not only of the target population but also of the different screening devices used in practice.

**Key Points:**

**Question** AI performance may vary across mammography devices, raising uncertainty about device-independent thresholds.

**Findings** AI scores differed considerably across devices, indicating the need for device-specific thresholds, but performance on one device remained poor.

**Critical relevance** The performance of an AI algorithm in mammography screening depends on adequately defined decision thresholds, and radiologists must be aware that AI scores can vary across mammography devices to avoid interpretation biases and to maintain diagnostic accuracy.

**Graphical Abstract:**

**Figure:**
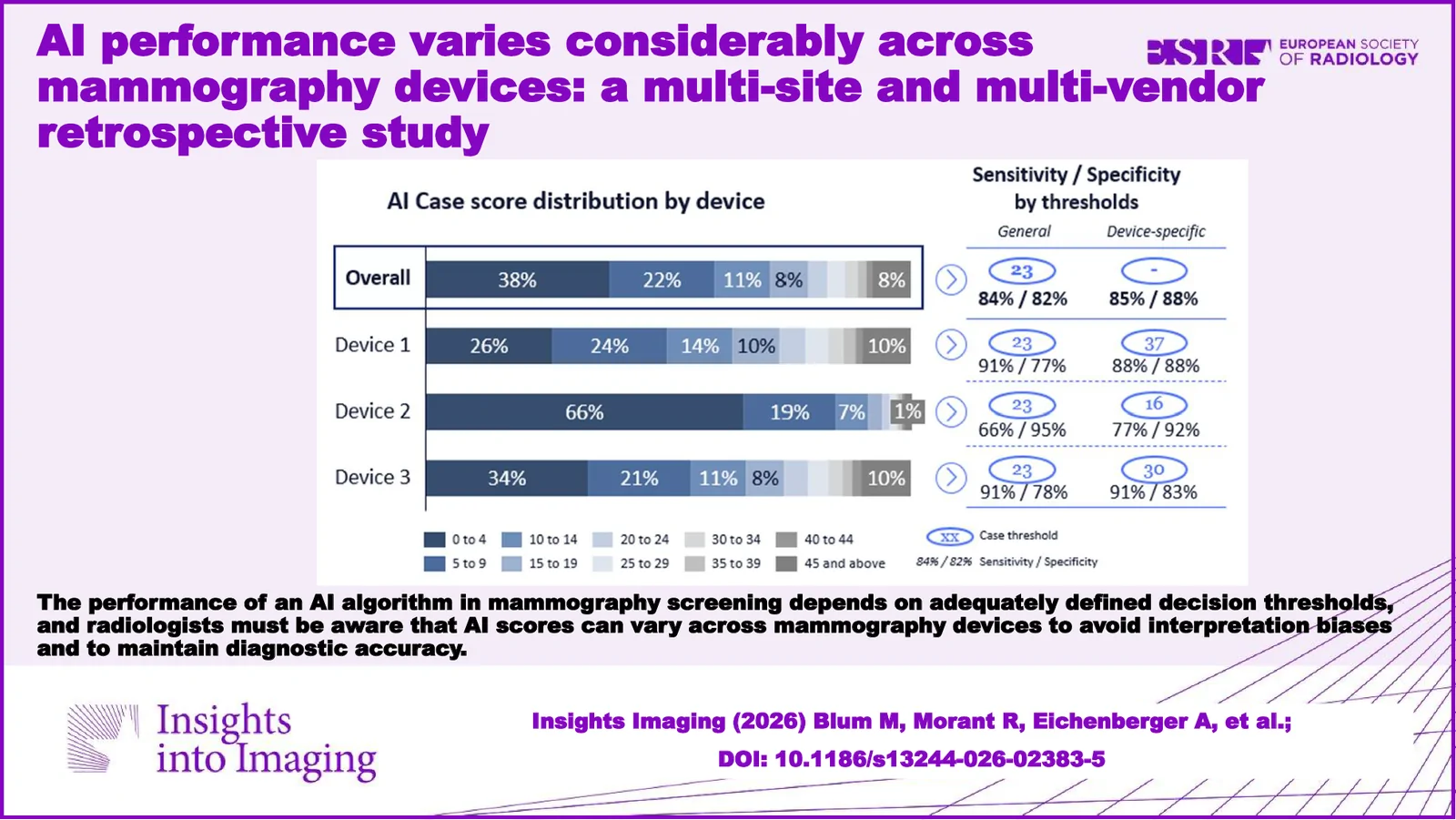

## Introduction

Organized mammography screening programs (MSPs) are a widely used public health strategy to reduce the burden of breast cancer [1, 2]. While the current practice of double reading increases the cancer detection rate (CDR) [3], it also imposes a considerable workload on radiologists [4]. Using artificial intelligence (AI) might reduce the workload of radiologists without compromising screening performance [5–9].

While various AI implementation scenarios in MSPs exist [10, 11], calibrating and setting thresholds for flagging suspicious mammographies remain critical tasks. Most thresholds for triaging mammographies as suspicious or unsuspicious aim to balance sensitivity (i.e., detecting cancer) and specificity (i.e., correctly identifying cancer-free cases) [12]. Safe AI implementation also depends on adequately training the algorithm using mammographies from the devices employed in screening programs. AI algorithms developed on non-representative datasets may lead to reduced diagnostic accuracy and adverse outcomes for screening participants [13].

Despite the growing number of retrospective and prospective studies assessing AI screening performance, most investigations rely on evaluating an AI algorithm at one screening site with a single mammography device from a single vendor [6–8, 14, 15], which limits the generalizability of the reported performance across screening sites and mammography devices. Studies comparing AI performance across different mammography devices, software, and screening sites report varying results in terms of sensitivity and specificity when AI was used as a standalone decision-maker [16–19]. However, these studies either used a single mammography device [16], tested an in-house-built AI algorithm [17], applied an AI algorithm to two distinct screening populations [18], or evaluated an AI algorithm on devices that were also part of its training dataset and lacked follow-up information on interval breast cancer (IBC) cases [19]. As multiple mammography devices across screening sites can be used in MSPs, evaluating AI screening performance across mammography devices remains an unaddressed yet very relevant question for clinical practice.

To address this research gap, we retrospectively evaluated a commercially available AI algorithm, trained on a globally diverse population, in a real-world setting using data from a Swiss MSP with six screening sites and three different mammography devices. We address the relevance of identifying and setting optimal thresholds and the potential consequences of inadequate calibration. Specifically, we assess how AI screening performance varies across mammography devices, whether device-specific thresholds improve AI screening performance, and whether such thresholds are sufficient to maintain safe diagnostic accuracy.

## Materials and methods

### Ethics approval

This retrospective research project was submitted to and approved by the responsible ethics committee (EKOS 24/132, ID 2024-01310). Participants in the screening program independently need to give their consent for using their anonymized data for statistical analyses. The study followed the STARD guideline for reporting diagnostic accuracy studies [20].

### Data sources, data processing, and AI algorithm

Data originates from participants within the Swiss canton of St. Gallen, who participated in the MSP “donna” in 2022 and 2023. The dataset includes information on the initial assessment by the two radiologists and on the consensus conference outcome, if one occurred, i.e., when at least one reader assessed the mammography as BI-RADS R3 or higher. Breast density was assessed subjectively by the radiologists. For our study, we used the final breast density assessment, that is, the consensus conference assessment, if one occurred, or the second reader’s assessment. All radiologists have multiple years of experience within and outside an MSP. Radiologists new to the program were accompanied by the medical chief of the MSP for quality assurance purposes. All radiologists undergo yearly continuing education courses. Linkage with the regional cancer registry using a unique screening identifier enabled the identification of 1-year IBC cases, i.e., diagnosed within 1 year after a mammography initially assessed as reviewed by radiologists. All study mammographies were retrospectively processed with ProFound AI® for FFDM/2D, version 3.1, by iCAD, Inc., configured with balanced (medium) average sensitivity and specificity optimization operation points. The computer-assisted detection and diagnosis AI algorithm, intended to be used concurrently by interpreting human readers, provides a numeric case score between 0 and 100 for each mammography. The case score reflects the certainty of the AI algorithm that the mammography contains a cancer case compared to an internal training database. The AI algorithm has no ability to assess prior mammographies. According to an iCAD, Inc. executive, the AI algorithm was trained on around 6 million images from over 100 screening sites in the US and across countries in Europe (including Switzerland), using mammographies acquired mostly on Siemens, GE, and Hologic mammography devices; however, no detailed information on the training dataset was published. The AI algorithm is Conformité Européenne marked.

### AI-integrated screening scenarios and threshold calibration

In our retrospective simulations, AI acts as a standalone decision-maker by flagging mammographies for suspiciousness of malignancy. For the calibration of optimal thresholds, we did not include a consensus conference decision, as it likely underestimates the ability of the AI algorithm to detect suspicious mammographies [15]. Consequently, in our simulations, participants with mammographies flagged by the AI algorithm are directly recalled for further investigations. In practice, such an approach, without the involvement of radiologists, would not be ethically and legally permissible [13, 21]. Therefore, we discuss the consequences of the number of AI-flagged mammographies on the workload of radiologists, especially in consensus conferences. Mammographies are flagged for suspiciousness by the AI algorithm when the AI case score is equal to or higher than the determined threshold. The optimal thresholds are determined using the Youden-Index [22], i.e., where sensitivity and specificity are maximized. Optimal thresholds were first determined using the combined dataset across all three mammography devices and then estimated separately for each device.

### Performance metrics and statistical analysis

The screening performance metrics in this retrospective study include sensitivity, specificity, and the corresponding average (balanced accuracy). Furthermore, the CDR and rate of IBCs are expressed as the number of cancers per 1000 mammographies. We assume that all cancer cases, including 1-year IBCs, were detected upon recall, i.e., flagged by AI, similar to [10]. The proportion of cases assessed by the AI as suspicious is labeled as “flagged by AI”.

For the AI case scores, the median score and the interquartile range (25–75%) are expressed. Kruskal–Wallis tests were used to assess differences in AI case score distribution across mammography devices. 95% confidence intervals (CIs) for screening performance metrics were calculated using the Clopper-Pearson “exact” method. Paired differences between scenarios or thresholds were compared using exact McNemar tests. The association between the mammography device and AI-assigned case score was further assessed using multivariable linear regression, adjusting for relevant participant and mammographic information. A *p*-value of less than 0.05 was considered statistically significant.

We conducted three sensitivity analyses. First, the mammography device with the highest reduction of screening performance was excluded. Second, a bootstrap approach with 10,000 replicates stratified for cancer status, i.e., each replicate containing the same number of cancer and non-cancer cases, was applied. The 2.5% and 97.5% percentile were reported as 95% confidence intervals to assess the certainty of the optimal threshold and the corresponding performance metrics. To support these bootstrap estimates, we also present the Youden-Index curve by threshold and the Receiver Operating Characteristic (ROC) curve across all three mammography devices and for each device separately. Third, we stratified the share of AI-flagged cases and CDR by radiologist-assessed breast density. All statistical analyses were done in Stata/BE version 18.0 (StataCorp LLC).

## Results

### Study population

**Fig. 1:**
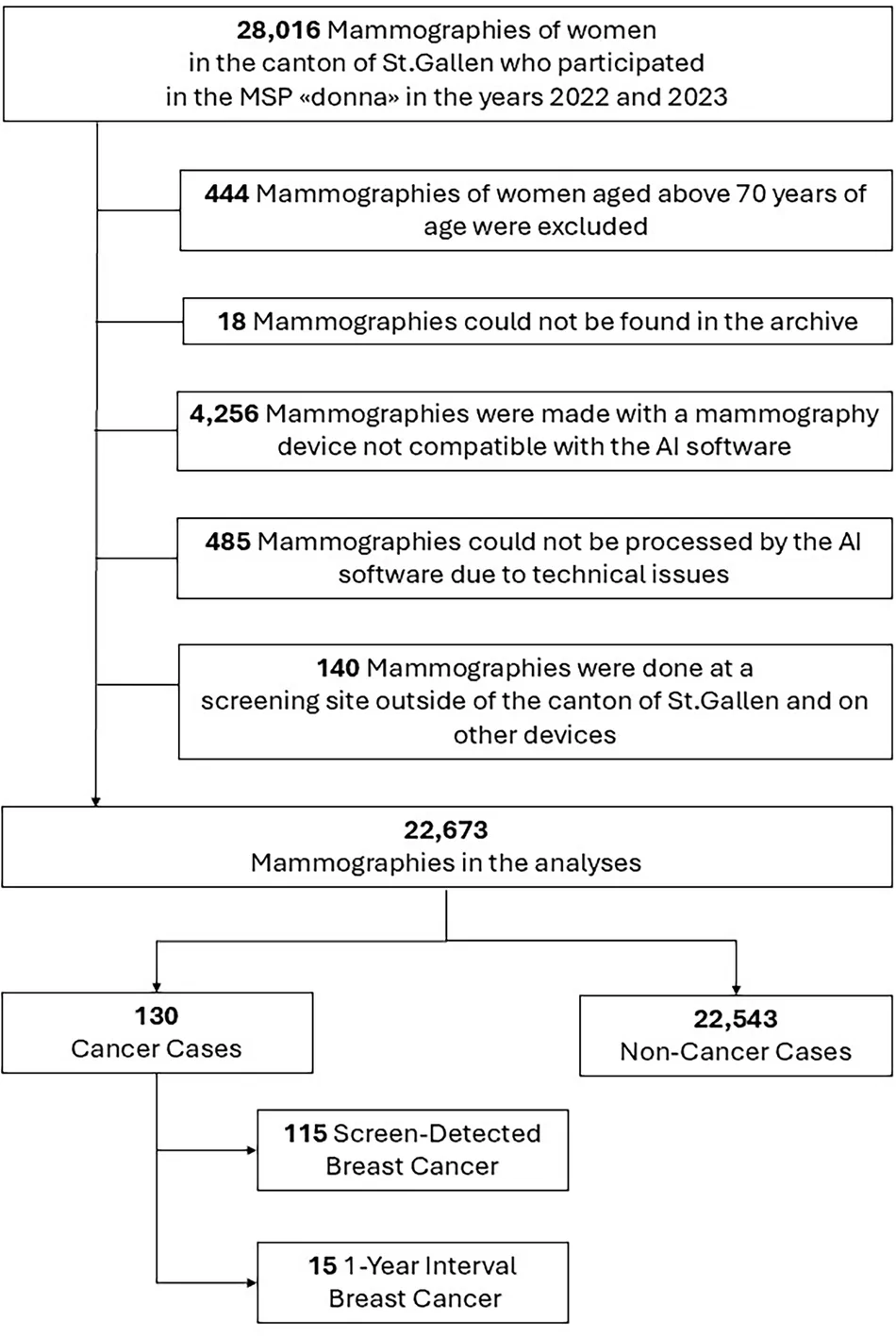
Derivation of the final dataset for the analyses

The initial sample for our study comprised 28,016 mammographies of residents of the Swiss canton of St. Gallen who participated in the MSP “donna” at one of its screening sites in the years of 2022 and 2023. For the study sample (Fig. 1), all mammographies of participants aged above 70 years of age (who participated upon request) were excluded (*N* = 444). Mammographies that could not be identified in the archive were excluded (*N* = 18). One screening site used a mammography device that was incompatible with the AI algorithm until September 2023; therefore, all examinations from this site were excluded (*N* = 4256). Other mammographies could not be processed by the AI algorithm (*N* = 485) due to other technical issues or incompleteness of images. Finally, participants who attended screening at an out-of-canton site using other mammography devices were also excluded (*N* = 140). The final sample of our study thus comprised 22,673 mammographies from six screening sites. Each site operated a single mammography device at any given time, although one site changed its device during the study period. Most mammographies were acquired on Siemens Mammomat Inspiration (11,773), followed by Philips MicroDose L50 (5764) and GE Senographe Pristina (5136) devices.

### Cohort characteristics

The mean age of the 22,673 screening participants in the final cohort was 58.8 years ± 5.8 (SD), with 21.0% participating for their prevalent screening (Table 1). 45.2% of the participants were assigned a high breast density (ACR c or d), 6.7% reported having had previous breast surgery, and 13.7% a known family history of breast cancer. Of the 22,673 mammographies, 6.3% were discussed in a consensus conference due to at least one of the two radiologists noticing an abnormality in the mammographic images. Following the conference, 3.1% of the women were recalled for further investigations. 115 screen-detected cancer (SDC) cases were included in the dataset and were diagnosed following recalls, resulting in a CDR of 5.07/1000 (95% CI: 4.19–6.09). Fifteen cases were diagnosed as a 1-year IBC, i.e., diagnosed within 1 year after a mammography assessed as unsuspicious. Initially, 6 of those IBCs were discussed in a consensus conference, and five were recalled for further investigations. A majority of mammographies were acquired on Siemens devices (51.9%), with the remaining mammographies acquired on Philips (25.4%) and GE (22.7%) devices.

**Table 1:** Descriptive statistics of participants’ characteristics and screening performance, overall and by each of the three mammography devices

	Overall	Siemens Inspiration	Philips L50	GE Pristina
Number of mammographies	22,673	11,773 (51.9%)	5764 (25.4%)	5136 (22.7%)
Age at screening*	58.8 (5.8)	59.1 (5.7)	58.8 (5.8)	58.4 (5.7)
Prevalent screenings	4772 (21.0%)	2321 (19.7%)	1237 (21.5%)	1214 (23.6%)
Breast density^†^				
ACR a	1969 (8.7%)	1210 (10.3%)	477 (8.3%)	282 (5.5%)
ACR b	10,455 (46.1%)	5664 (48.1%)	2549 (44.2%)	2242 (43.7%)
ACR c	9650 (42.6%)	4634 (39.4%)	2544 (44.1%)	2472 (48.1%)
ACR d	599 (2.6%)	265 (2.2%)	194 (3.4%)	140 (2.7%)
Reported family history of breast cancer	3098 (13.7%)	1759 (14.9%)	607 (10.5%)	732 (14.3%)
Previous breast surgery^‡^	1522 (6.7%)	837 (7.1%)	337 (5.8%)	348 (6.8%)
Consensus conference rate^§^	1425 (6.3%)	639 (5.4%)	472 (8.2%)	314 (6.1%)
Recall rate	706 (3.1%)	348 (3.0%)	215 (3.7%)	143 (2.8%)
SDC (CDR; 95% CI)	115 (5.07; 4.19–6.09)	62 (5.27; 4.04–6.75)	33 (5.73; 3.94–8.03)	20 (3.89; 2.38–6.01)
Tumor size*	19.9 (15.8)	18.9 (13.6)	21.1 (17.6)	20.8 (19.4)
Tumor stage				
In situ	27 (23.5%)	12 (19.4%)	9 (27.3%)	6 (30.0%)
I	48 (41.7%)	25 (40.3%)	16 (48.5%)	7 (35.0%)
II	34 (29.6%)	21 (33.9%)	7 (21.2%)	6 (30.0%)
III	3 (2.6%)	1 (1.6%)	1 (3.0%)	1 (5.0%)
IV	2 (1.7%)	2 (3.2%)	0	0
Unknown	1 (0.9%)	1 (1.6%)	0	0
Histological grading				
G1	11 (9.6%)	5 (8.1%)	4 (12.1%)	2 (10.0%)
G2	52 (45.2%)	30 (48.4%)	14 (42.4%)	8 (40.0%)
G3	23 (20.0%)	13 (21.0%)	6 (18.2%)	4 (20.0%)
Unknown	29 (25.2%)	14 (22.6%)	9 (27.3%)	6 (30.0%)
IBC (rate per 1000 screens; 95% CI)	15 (0.66; 0.37–1.01)	12 (1.02; 0.53–1.78)	2 (0.35; 0.04–1.25)	1 (0.19; 0.00–1.08)
Sensitivity (95% CI)	88.5% (81.7–93.4)	83.8% (73.4–91.3)	94.3% (80.8–99.3)	95.2% (76.2–99.9)
Specificity (95% CI)	97.4% (97.2–97.6)	97.6% (97.3–97.8)	96.9% (96.4–97.3)	97.6% (97.2–98.0)
Balanced accuracy (95% CI)	92.9% (89.4–95.5)	90.7% (85.3–94.6)	95.6% (88.6–98.3)	96.4% (86.7–98.9)

Percentage of the total mammographies/participants in brackets

*ACR* American College of Radiology, *SDC* screen-detected breast cancer, *CDR* cancer detection rate, *CI* confidence interval, *IBC* interval breast cancer

* We report the mean age/size in mm, with standard deviation in brackets

^†^ Breast density is assigned by radiologists according to the ACR Breast Imaging Reporting and Data System (BI-RADS)

^‡^ Previous breast surgeries are self-reported by women prior to their mammography and may include breast enlargement or reduction, as well as surgeries due to benign or malignant changes in the breast. Women with previous breast cancer may opt to participate in the program

^§^ Consensus conferences are held when at least one of the two radiologists detects abnormalities in the mammography

In the initial screening without AI, an overall sensitivity of 88.5% (95% CI: 81.7–93.4%) and a specificity of 97.4% (95% CI: 97.2–97.6%) were achieved. Balanced accuracy, i.e., the average of sensitivity and specificity, was 92.9% (95% CI: 89.4–95.5%) overall and ranged from 90.7% (Siemens) to 96.4% (GE). Minor differences in characteristics of the participants and screening performance can be seen among the six screening sites (shown in eTable 1).

### AI assessment

**Fig. 2:**
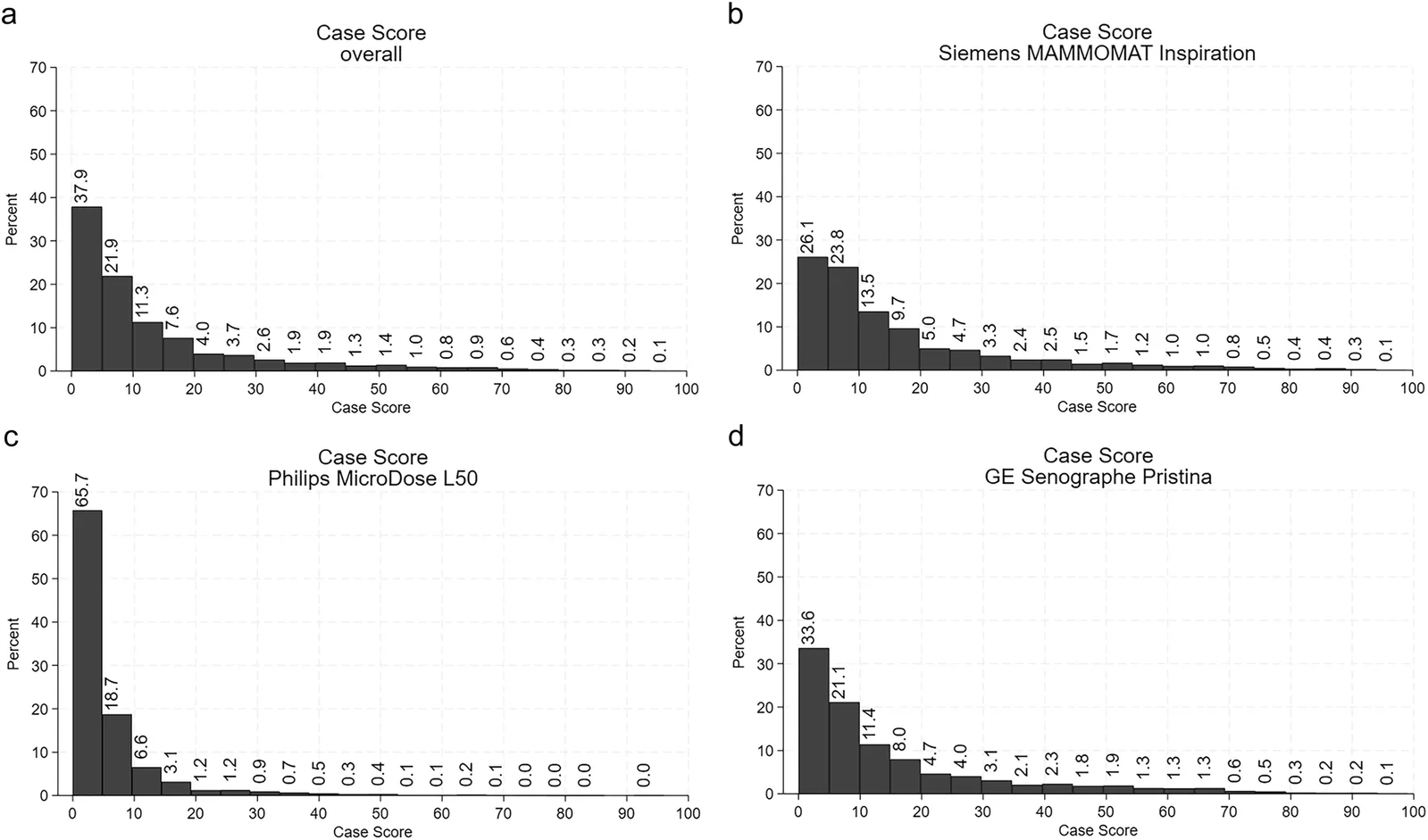
AI case score distribution for each mammography device. **a** For all mammographies, **b** mammographies acquired on Siemens MAMMOMAT Inspiration, **c** mammographies acquired on Philips MicroDose L50, and **d** mammographies acquired on GE Senographe Pristina

Figure 2 shows the case score distribution overall (Fig. 2a), and for each mammography device (Fig. 2b–d). While the overall median case score was 7 (25–75%: 3–17), distribution differences are evident when case scores are portrayed for each mammography device separately. Mammographies acquired on the Siemens device had a median case score of 10 (25–75%: 4–21), whereas the median case score of GE mammographies was 8 (25–75%: 4–20). The median case score of mammographies acquired on the Philips device was 3 (25–75%: 2–6), and thus, significantly lower (*p* < 0.001) than the case scores from the other two devices. More specifically, 28.1% of all Philips mammographies had a case score of 1 or 2, 24.8% of 3 and 12.9% of 4. In multivariable linear regression analysis (eTable 2), adjusting for breast density, age, prevalent screening, family history of breast cancer, and previous breast surgery, the mammography device was significantly associated with the AI-assigned case score. The adjusted mean case score for mammographies acquired on Philips devices was 6.41, approximately 10 points lower than those acquired on Siemens (16.60) and GE (15.79) devices (both pairwise *p* < 0.001). The 115 SDC cases were retrospectively assessed with a median case score of 67 (25–75%: 37–85), with Siemens and GE SDCs having a significantly higher median case score of 75 (25–75%: 56–86) and 74 (25–75%: 52.5–93), respectively, than Philips SDC cases with a median of 28 (25–75%: 16–45) (*p* < 0.001) as shown in eFigure 1. Of the 15 1-year IBC cases, 12 were diagnosed after a mammography on a Siemens device initially reviewed as unsuspicious by radiologists, with a median case score of 43.5 (25–75%: 13–55.5).

### AI performance on a general case score threshold

**Fig. 3:**
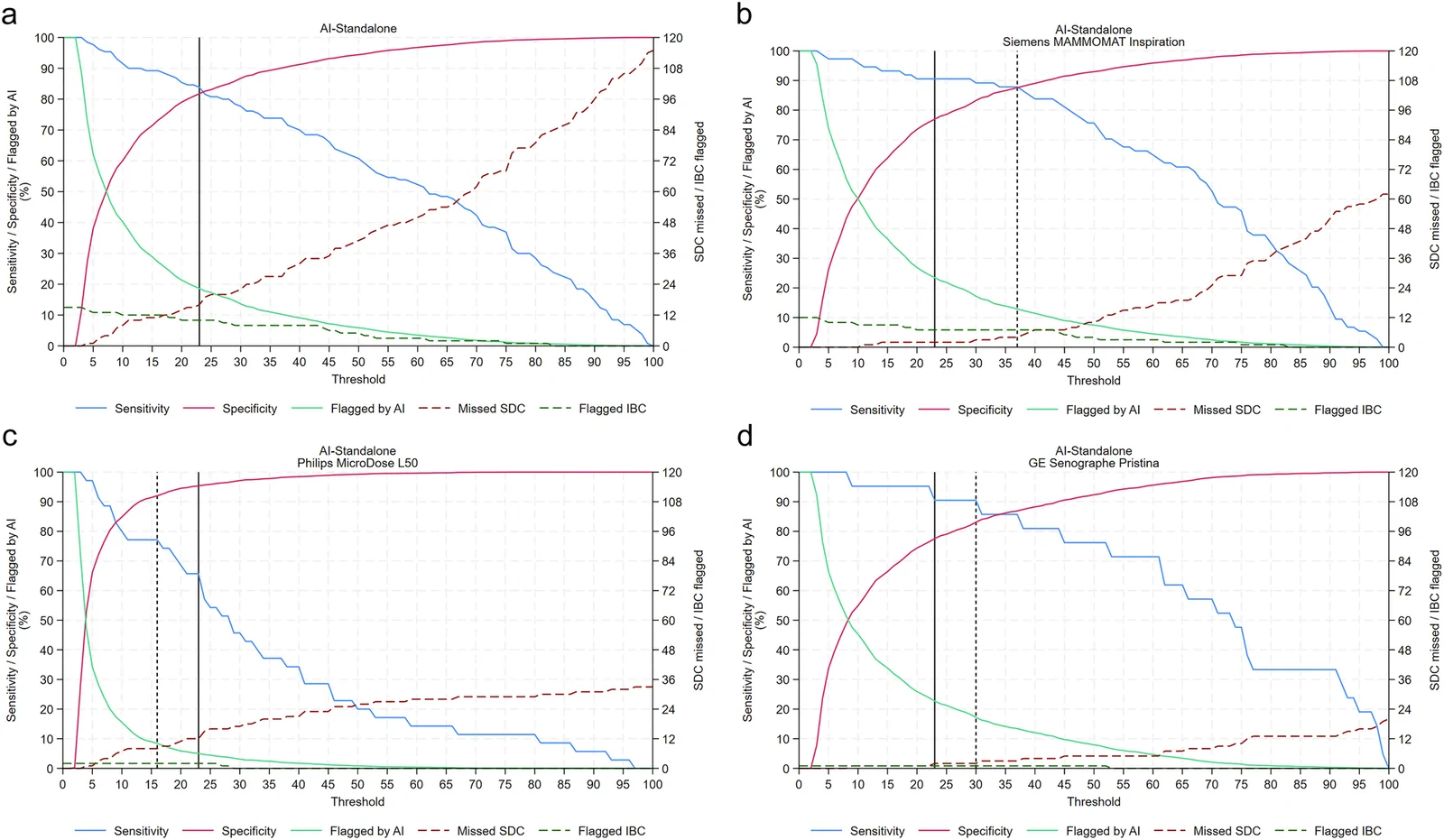
AI-standalone threshold calibration for each mammography device. **a** For all mammographies, **b** mammographies acquired on Siemens MAMMOMAT Inspiration, **c** acquired on Philips MicroDose L50, and **d** acquired on GE Senographe Pristina; solid vertical line represents the optimal general threshold at cutoff 23, whereas the dashed vertical lines in **b**–**d** represent the optimal device-specific thresholds. AI, artificial intelligence; SDC, screen-detected breast cancer; IBC, interval breast cancer

Overall, the optimal case score threshold is determined to be at the cutoff of 23, where according to the Youden-Index sensitivity and specificity are maximized (Fig. 3a). Using this threshold, all cases with a case score of 23 or higher were flagged by the AI algorithm, resulting in 18.6% of cases being flagged overall, with proportions ranging from 5.0% (Philips) to 23.5% (Siemens), as shown in Table 2a. This general threshold resulted overall in a sensitivity of 83.8% (95% CI: 76.4–89.7%) and a specificity of 81.8% (95% CI: 81.2–82.3%). The corresponding average (balanced accuracy) was 82.8% (95% CI: 78.8–85.9%) and ranged from 80.5% (Philips) to 84.0% (GE). The simulated CDR was 4.81/1000 (95% CI: 3.95–5.80), which was not significantly lower than in the standard double-reading with consensus conference (*p* = 0.33). However, 16 out of 115 SDC cases would not have been flagged by the AI algorithm (12 on the Philips, and 2 each on the Siemens and GE devices), whereas 10 out of 15 IBC cases would have been flagged (7 on Siemens, 2 on Philips, and 1 on GE). Although the difference was not significant, the Philips device showed a notable reduction in both sensitivity and CDR. For instance, the CDR without AI was 5.73/1000 (95% CI: 3.94–8.03), and 3.99/1000 (95% CI: 2.53–5.98) when applying AI with a general threshold.

**Table 2:** AI performance on general threshold and device-specific thresholds

	(a) General threshold				(b) Device-specific-threshold			
	Overall	Siemens Inspiration	Philips L50	GE Pristina	Overall	Siemens Inspiration	Philips L50	GE Pristina
Mammographies	22,673	11,773	5764	5136	22,673	11,773	5764	5136
SDC	115	62	33	20	115	62	33	20
IBC	15	12	2	1	15	12	2	1
Threshold	23	23	23	23	-	37	16	30
Flagged by AI	18.6%	23.5%	5.0%	22.7%	12.7%	12.9%	8.4%	17.2%
Sensitivity (95% CI)	83.8% (76.4–89.7)	90.5% (81.5–96.1)	65.7% (47.8–80.9)	90.5% (69.6–98.8)	85.4% (78.1–91.0)	87.8% (78.2–94.3)	77.1% (59.9–89.6)	90.5% (69.6–98.8)
Specificity (95% CI)	81.8% (81.2–82.3)	76.9% (76.2–77.7)	95.4% (94.8–95.9)	77.6% (76.4–78.7)	87.7% (87.2–88.1)	87.6% (87.0–88.2)	92.0% (91.3–92.7)	83.1% (82.0–84.1)
Balanced accuracy (95% CI)	82.8% (78.8–85.9)	83.7% (78.8–86.9)	80.5% (71.3–88.4)	84.0% (73.0–88.8)	86.5% (82.7–89.5)	87.7% (82.6–91.2)	84.6% (75.6–91.1)	86.8% (75.8–91.5)
SDC	99	60	21	18	101	58	25	18
IBC	10	7	2	1	10	7	2	1
CDR (95% CI)	4.81 (3.95–5.80)	5.69 (4.41–7.22)	3.99 (2.53–5.98)	3.70 (2.23–5.77)	4.90 (4.03–5.89)	5.52 (4.26–7.03)	4.68 (3.09–6.81)	3.70 (2.23–5.77)

(a) Screening performance on general threshold, (b) screening performance on device-specific thresholds

*SDC* screen-detected breast cancer, *IBC* interval breast cancer, *AI* artificial intelligence, *CI* confidence interval, *CDR* cancer detection rate

### AI performance on device-specific thresholds

The optimal threshold varies considerably among the mammography devices, with the Philips device having an optimal threshold of 16, the GE device of 30, and the Siemens device of 37 (c.f. Fig. 3b–d). Using these device-specific thresholds, the proportions of flagged mammographies by the AI algorithm was 12.7%, ranging from 8.4% (Philips) to 17.2% (GE), as shown in Table 2b. Consequently, by using device-specific thresholds, the proportion of mammographies flagged by the AI algorithm was overall reduced by almost one-third from 18.6% to 12.7% (*p* < 0.001). The overall sensitivity was 85.4% (95% CI: 78.1–91.0%) and the specificity 87.7% (95% CI: 87.2–88.1%). The balanced accuracy was 86.5% (95% CI: 82.7–89.5%), ranging from 84.6% (Philips) to 87.7% (Siemens). The use of device-specific thresholds resulted overall in a slightly higher CDR of 4.90/1000 (95% CI: 4.03–5.89) than using a general threshold (*p* = 0.69). While the number of flagged IBCs remained stable at 10 cases, the number of flagged SDC mammographies increased by two, leaving 14 SDC cases undetected by the AI algorithm. Device-specific thresholds detected four SDC cases that remained undetected with a general threshold. Conversely, two SDC cases were flagged only with a general threshold.

### Sensitivity analyses

While the overall screening performance of the AI algorithm as a standalone decision-maker for Siemens and GE devices was close to the current status quo performance (double read with consensus conference), the performance on Philips devices was considerably lower. More specifically, even with device-specific thresholds, the CDR on Philips mammographies was 4.68/1000 (95% CI: 3.09–6.81). This considerable reduction also affected the overall screening performance using all three devices. The CDR was 5.07 (95% CI: 4.19–6.09) in the standard of care and 4.90/1000 (95% CI: 4.03–5.89) with device-specific thresholds. In a sensitivity analysis, shown in eTable 3, the Philips device was excluded from the analyses. Standard of care without AI resulted in a CDR of 4.85/1000 (95% CI: 3.86–6.02), with AI as a standalone decision-maker with a general threshold yielded a CDR of 4.91/1000 (95% CI: 3.91–6.08), and with a device-specific threshold a CDR of 4.97/1000 (95% CI: 3.96–6.15). Screening performance metrics using 10,000 stratified bootstrap samples show consistent and similar results (shown in eTable 4). eFigure 2 and eFigure 3 show the Youden-Index by threshold and the Receiver Operating Characteristic (ROC) curve, respectively, across all three mammography devices and for each device separately. Lastly, eTable 5 presents the proportion of AI-flagged mammographies and the CDR using a general AI threshold and device-specific thresholds, stratified by radiologist-assessed breast density. The table also reports on the proportion of consensus conference discussions and the recall rate under the current double-reading process without AI.

## Discussion

We aimed to retrospectively assess the performance of an AI algorithm as a standalone decision-maker on mammographies acquired on three different mammography devices, with a focus on identifying and setting optimal threshold(s). More specifically, we evaluated whether device-specific thresholds enhance screening performance and whether they are sufficient to maintain diagnostic accuracy. Using device-specific thresholds, the number of AI-flagged mammographies dropped by nearly one-third and enhanced screening performance. Consequently, since in reality mammographies are discussed at consensus conferences, the number of cases and the workload of radiologists can be considerably reduced, while maintaining or improving CDR. However, the share of cases for consensus conference discussions is still increased by at least a factor of two compared to the current screening standard in the MSP “donna,” raising questions about whether AI-based screening reduces the overall workload [23]. This might particularly occur if these AI-flagged cases are difficult to interpret. In our study, the overall screening performance with and without device-specific thresholds was reduced compared to the current double-reading with consensus conferences, adding to mixed evidence of AI performance as a standalone decision-maker [6, 11, 12, 17, 24].

Identifying and setting optimal thresholds remain crucial tasks for an MSP [12], as screening performance of AI algorithms might vary across mammography devices and software versions [16–19]. Using device-specific thresholds in our study considerably improved screening performance, consistent with other findings reported [18]. Even once determined, the performance of AI algorithms requires continuous monitoring [25], as software updates to the mammography equipment [16] or to the AI algorithm itself [26] may affect image outputs and interpretations. Monitoring becomes particularly critical when the AI algorithm has not been trained on the specific mammography devices used in practice [27, 28]. In our study, the AI algorithm generated a notably different distribution of case scores for one of the mammography devices. For this device, even the application of a device-specific threshold would have substantially reduced screening performance.

We hypothesize that reduced AI performance results from limited device-specific training, indicating that the AI algorithm in our study is not yet fully calibrated for this mammography device and requires further training or adaptation before safe deployment. However, other factors may also explain why this device performs differently, such as the different interpretation of the AI algorithm on “for-presentation” versus “for-processing” (raw) images [29], variations in image quality (e.g., due to positioning) [30], or other technical acquisition factors [31]. All these factors highlight the importance of pre-deployment testing and validation of the AI algorithm on local data, which may include different mammography devices. Consequently, without adequate calibration, diagnostic accuracy could be reduced, potentially impacting screening participants negatively, i.e., false-positive recall or missed cancer [13].

Device-specific thresholds, however, present several practical challenges for the organization of an MSP and for radiologists interpreting AI scores. Different thresholds for each mammography device and/or screening site might challenge an efficient technical implementation. Furthermore, since radiologists typically read mammographies acquired on different devices, interpretation biases could occur if the meaning of AI case scores varies. Primarily, if one device has a considerable different AI case score distribution, diagnostic accuracy may be compromised if radiologists are not aware of this different distribution. This could lead to automation and confirmation biases [32], potentially leading to increased false-positive recalls and/or increased false-negative findings, resulting in missed or delayed cancer diagnoses [33]. More specifically, low case scores might lead to the erroneous dismissal of suspicious findings. Thus, it is crucial to raise awareness among radiologists involved in the screening program of the variability in AI-assigned case scores across different mammography devices to mitigate the risk of interpretation biases [34].

Our retrospective study has five limitations. First, human-AI interaction and consensus-conference decision-making cannot be simulated, thus making the estimated CDR hypothetical. Second, we observed notable differences in CDR across mammography devices in the current setting with no AI use, for which we have no clear explanation, as screening setting, approach to reading, and radiologists’ experience are standardized and controlled for within the MSP. Technical attributes (e.g., different detector techniques across devices) or different radiation doses might be associated with varying image acquisition, processing, and output. These variations could lead to differences in image quality (e.g., image resolution or pixel size), which might explain the underlying screening performance differences with and without AI. Third, our data include subjective breast density assessment by radiologists, which might explain the comparatively low proportion of extremely dense breasts (i.e., ACR d) and therefore limit the generalizability of our findings. Fourth, we only consider 1-year IBCs, as 2-year IBCs were not yet fully available for our study sample. However, first-year IBCs have been shown to be more likely false-negatives within MSPs [35]. Fifth, the small sample size, particularly the small number of cancer cases, may limit the generalizability and robustness of our results, although the data originate from a real-world screening program rather than a cancer-enriched dataset. The small sample size also precluded performing a split-sample validation of the determined thresholds.

Our research with real-world data from regular MSP participants encourages programs and radiologists to carefully determine and interpret AI scores and thresholds for each mammography device separately. Although the AI algorithm in this study was licensed for all mammography devices, remarkable differences in case score distributions—initially unknown to the MSP and radiologists—became evident. These variances can significantly alter (lower) real-life screening performance, especially when radiologists read mammographies acquired on different devices. Our results emphasize the necessity to constantly observe AI-supported screening performance. If necessary, AI thresholds must be adjusted, or the AI algorithm should not be used on a particular device.

From a practical perspective, using a single threshold may be preferable given the technical and interpretational challenges. Our findings could also encourage the harmonization of mammography devices across screening sites within an MSP. AI providers, on the other hand, are asked to disclose detailed information about their training dataset, including the mammography devices used and the demographic characteristics of the screened individuals (e.g., age range, race, ethnicity). This information can help MSPs and radiologists in making informed decisions when purchasing or applying an AI algorithm on their mammography devices.

## Electronic Supplementary Material

Supplementary information

## Data Availability

Fully anonymized dataset available upon reasonable request.

## Abbreviations

ACR: American College of Radiology
AI: Artificial intelligence
BI-RADS: Breast Imaging Reporting and Data System
CDR: Cancer detection rate (per 1000 mammographies)
CI: Confidence interval
IBC: Interval breast cancer
MSP: Mammography screening program
ROC: Receiver operating characteristic
SDC: Screen-detected breast cancer
