# Supplementary material for: AI performance varies considerably across mammography devices: a multi-site and multi-vendor retrospective study
Source: Insights Imaging. 2026 Aug 26;17:209. doi: 10.1186/s13244-026-02383-5 (PMC13518669; doi:10.1186/s13244-026-02383-5)
Supplement: Supplementary file 1 [file 13244_2026_2383_MOESM1_ESM.pdf]

AI Performance varies considerably across Mammography Devices: A Multi-Site and Multi-Vendor Retrospective Study

Electronic Supplementary Material

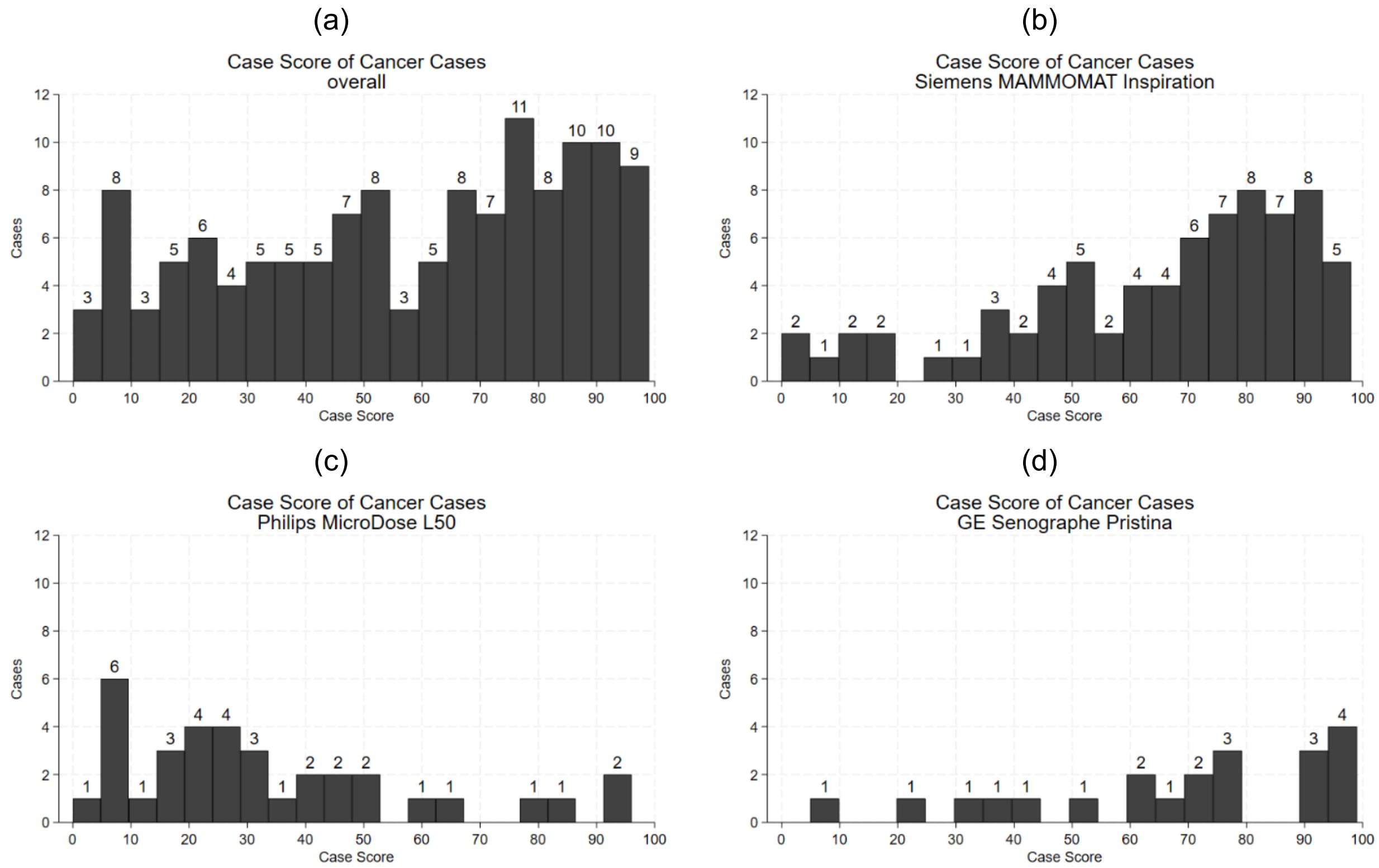

eFigure 1: AI Case Scores of Cancer Cases overall, and for each Mammography Device

Notes: (a) for all mammographies, (b) mammographies acquired on Siemens MAMMOMAT Inspiration, (c) mammographies acquired on Philips MicroDose L50, and (d) mammographies acquired on GE Senographe Pristina.

eFigure 2: AI-Standalone Performance according to the Youden-Index across all three Mammography Devices and for each Device separately

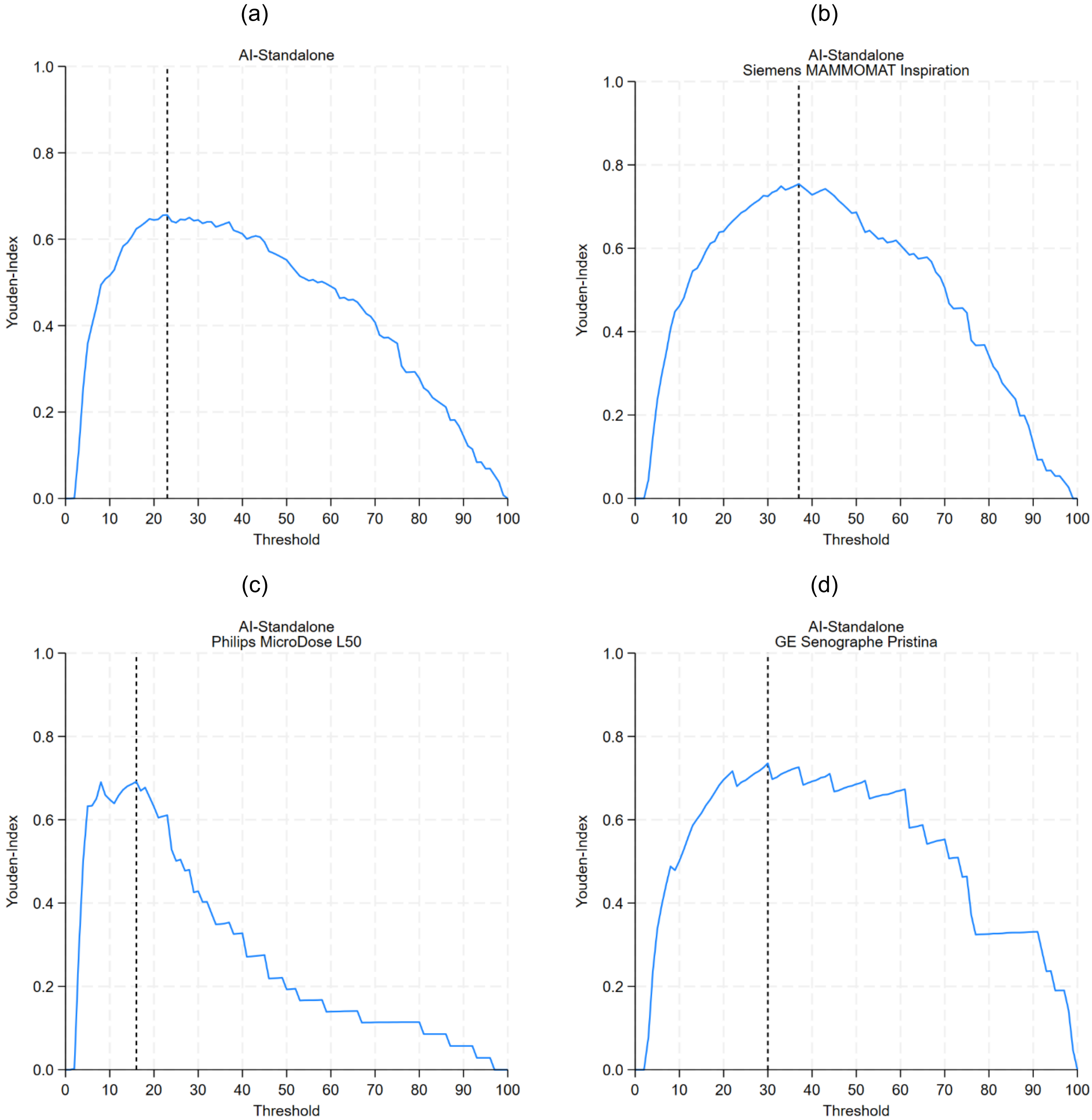

Notes: The Youden-Index on the Y-axis displays the score of (Sensitivity + Specificity – 1) for each Threshold portrayed on the X-axis. The vertical dashed line represents the optimal determined threshold with the highest Youden-Index. (a) for all mammographies, (b) mammographies acquired on Siemens MAMMOMAT Inspiration, (c) mammographies acquired on Philips MicroDose L50, and (d) mammographies acquired on GE Senographe Pristina.

eFigure 3: AI-Standalone Performance displayed as Receiver Operating Characteristic (ROC) Curve across all three Mammography Devices and for each Device separately

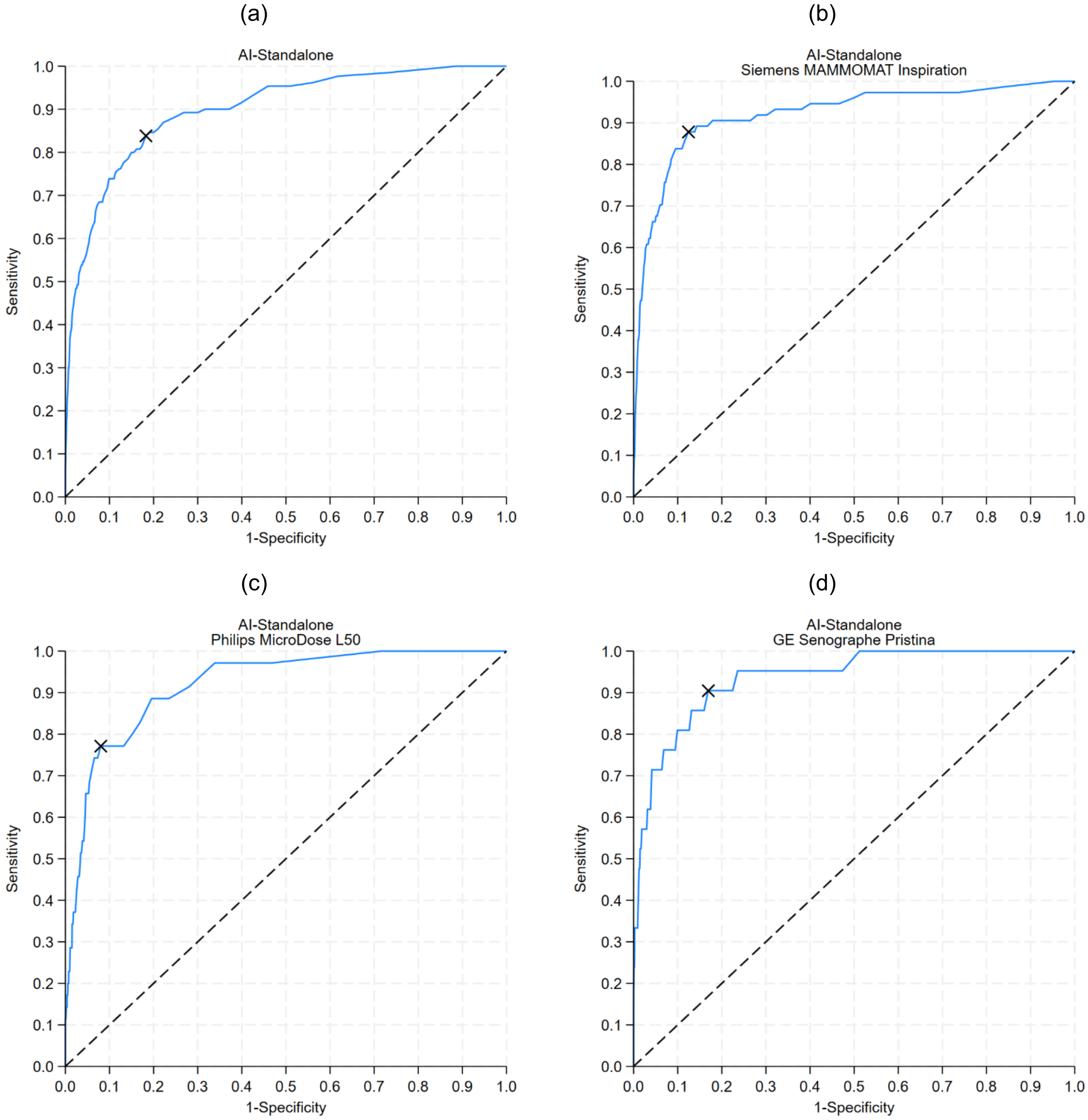

Notes: The graphs show the Receiver Operating Characteristic (ROC) Curve, with Sensitivity plotted on the Y-Axis and 1-Specificity on the X-Axis. The X symbol marks the location of the optimal determined threshold according to the Youden-Index. (a) for all mammographies, (b) mammographies acquired on Siemens MAMMOMAT Inspiration, (c) mammographies acquired on Philips MicroDose L50, and (d) mammographies acquired on GE Senographe Pristina.

eTable 1: Descriptive Statistics (Participants' Characteristics and Screening Performance) for each of the six screening sites

	Screening Site 1	Screening Site 2	Screening Site 3	Screening Site 4	Screening Site 3	Screening Site 5	Screening Site 6
Mammography device	Siemens	Siemens	Siemens	Philips	GE	GE	GE
Number of mammographies	6,259	2,901	2,613	5,764	2,965	1,802	369
Age at screening *	59.2 (5.7)	59.2 (5.7)	58.8 (5.8)	58.8 (5.8)	58.3 (5.7)	58.7 (5.8)	57.6 (6.0)
Prevalent screenings	1,145 (18.3%)	593 (20.4%)	583 (22.3%)	1,237 (21.5%)	664 (22.4%)	445 (24.7%)	105 (28.5%)
Breast density†							
- ACR a	839 (13.4%)	30 (1.0%)	341 (13.1%)	477 (8.3%)	264 (8.9%)	2 (0.1%)	16 (4.3%)
- ACR b	2,936 (46.9%)	1,593 (54.9%)	1,135 (43.4%)	2,549 (44.2%)	1,336 (45.1%)	772 (42.8%)	134 (36.3%)
- ACR c	2,361 (37.7%)	1,257 (43.3%)	1,016 (38.9%)	2,544 (44.1%)	1,242 (41.9%)	1,022 (56.7%)	208 (56.4%)
- ACR d	123 (2.0%)	21 (0.7%)	121 (4.6%)	194 (3.4%)	123 (4.1%)	6 (0.3%)	11 (3.0%)
Reported family history of breast cancer	950 (15.2%)	452 (15.6%)	357 (13.7%)	607 (10.5%)	431 (14.5%)	236 (13.1%)	65 (17.6%)
Previous breast surgery	441 (7.0%)	216 (7.4%)	180 (6.9%)	337 (5.8%)	202 (6.8%)	110 (6.1%)	36 (9.8%)
Consensus conference rate‡	375 (6.0%)	153 (5.3%)	111 (4.2%)	472 (8.2%)	159 (5.4%)	107 (5.9%)	48 (13.0%)
Recall rate	206 (3.3%)	94 (3.2%)	48 (1.8%)	215 (3.7%)	70 (2.4%)	54 (3.0%)	19 (5.1%)
SDC (CDR; 95%-CI)	37 (5.91; 4.17-8.14)	17 (5.86; 3.42-9.37)	8 (3.06; 1.32-6.02)	33 (5.73; 3.94-8.03)	12 (4.05; 2.09-7.06)	5 (2.77; 0.90-6.46)	3 (8.13; 1.68-2.36)
IBC (Rate per 1,000 screens; 95%-CI)	7 (1.12; 0.45-2.30)	4 (1.38; 0.38-3.53)	1 (0.38; 0.01-2.13)	2 (0.35; 0.04-1.25)	1 (0.34; 0.01-1.88)	0 (0; 0-2.00)	0 (0; 0-9.95)
Sensitivity (95%-CI)	84.1% (69.9-93.4)	81.0% (58.1-94.6)	88.9% (51.8-99.7)	94.3% (80.8-99.3)	92.3% (64.0-99.8)	100% (47.8-100)	100% (29.2-100)
Specificity (95%-CI)	97.3% (96.9-97.7)	97.4% (96.7-97.9)	98.5% (97.9-98.9)	96.9% (96.4-97.3)	98.1% (97.5-98.5)	97.3% (96.4-98.0)	95.6% (93.0-97.5)
Balanced accuracy (95%-CI)	90.7% (83.4-95.6)	89.2% (77.4-96.3)	93.7% (74.9-99.3)	95.6% (88.6-98.3)	95.2% (80.8-99.2)	98.7% (72.1-99.0)	97.8% (61.1-98.8)

*Notes: Percentage of the total mammographies/participants in brackets. * We report the mean age, with standard deviation in brackets. † Breast density is assigned by radiologists. ‡Consensus conferences are held when at least one of the two radiologists detected abnormalities in mammography. Screening site 3 changed their mammography device in the study period. Abbreviations: ACR = American College of Radiology, SDC = Screen-detected breast cancer, CDR = Cancer detection rate, CI = Confidence interval, IBC = Interval breast cancer*

eTable 2: Multivariable linear regression of AI-assigned case score on mammography device and patient characteristics (N = 22,673)

Number of obs	22,673
F(9, 22663)	359.32
Prob > F	0
R-squared	0.0735
Root MSE	16.044

**AI-assigned case_score**	**Coefficient**	**Robust std. err.**	**t**	**P > |t|**	**95% CI**		
Device (Reference: Siemens)							
Philips	-10.185	0.201	-50.71	0.00	-10.578	-9.791	***
GE	-0.811	0.304	-2.66	0.01	-1.407	-0.214	***
Breast Density (Reference: ACR a)							
ACR b	2.695	0.354	7.61	0.00	2.001	3.389	***
ACR c	2.751	0.359	7.66	0.00	2.046	3.455	***
ACR d	1.507	0.693	2.17	0.03	0.148	2.865	**
Age	0.004	0.022	0.16	0.87	-0.040	0.047	
Prevalent Screening	0.120	0.312	0.38	0.70	-0.491	0.731	
Family History	0.908	0.325	2.80	0.01	0.272	1.544	***
Previous Breast Surgery	3.194	0.463	6.90	0.00	2.286	4.102	***
Constant	13.571	1.410	9.62	0.00	10.807	16.335	***

*Notes: Multilinear linear regression analysis using the numeric AI-assigned case score as dependent variable / outcome. The following mammography and participant information were used as independent variables: breast density (ACR a-d; assessed by the radiologists), numeric age of the woman at the time of the mammography, binary information whether mammography was a prevalent screening, i.e., first-time participation in the screening program, women had a known family history of breast cancer (self-reported), and whether the woman had a previous breast surgery. Adjusted for the cofounding information, the mean case score for three mammography devices were as follows and were significantly pairwise different: Siemens = 16.60, Philips = 6.41, GE = 15.79. Abbreviations: ACR = American College of Radiology. Statistical significance levels: * p<0.1; ** p<0.05; *** p<0.01*

eTable 3: AI Performance on General Threshold and Device-Specific Thresholds without the Philips device

	(a) Screening Performance without AI			(b) General Threshold			(c) MGD-Specific-Threshold		
	Overall	Siemens Inspiration	GE Pristina	Overall	Siemens Inspiration	GE Pristina	Overall	Siemens Inspiration	GE Pristina
Mammographies<br>- SDC<br>- IBC	16,909<br>82<br>13	11,773<br>62<br>12	5,136<br>20<br>1	16,909<br>82<br>13	11,773<br>62<br>12	5,136<br>20<br>1	16,909<br>82<br>13	11,773<br>62<br>12	5,136<br>20<br>1
Threshold	-	-	-	37	37	37	-	37	30
Flagged by AI	-	-	-	13.1%	12.9%	13.4%	14.2%	12.9%	17.2%
Sensitivity (95%-CI)	86.3% (77.7-92.0)	83.8% (73.4-91.3)	95.2% (76.2-99.9)	87.4% (79.0-93.3)	87.8% (78.2-94.3)	85.7% (63.7-97.0)	88.4% (80.2-94.1)	87.8% (78.2-94.3)	90.5% (69.6-98.8)
Specificity (95%-CI)	97.6% (97.3-97.8)	97.6% (97.3-97.8)	97.6% (97.2-98.0)	87.4% (86.9-97.9)	87.6% (87.0-88.2)	86.9% (85.9-87.8)	86.2% (85.7-86.7)	87.6% (87.0-88.2)	83.1% (82.0-84.1)
Balanced Accuracy (95%-CI)	92.0% (87.5-94.9)	90.7% (85.3-94.6)	96.4% (86.7-98.9)	87.4% (82.9-90.6)	87.7% (82.6-91.2)	86.3% (74.8-92.4)	87.3% (83.0-90.4)	87.7% (82.6-91.2)	86.8% (75.8-91.5)
SDC	82	62	20	75	58	17	76	58	18
IBC	-	-	-	8	7	1	8	7	1
CDR (95%-CI)	4.85 (3.86-6.02)	5.27 (4.04-6.75)	3.89 (2.38-6.01)	4.91 (3.91-6.08)	5.52 (4.26-7.03)	3.50 (2.08-5.53)	4.97 (3.96-6.15)	5.52 (4.26-7.03)	3.70 (2.23-5.77)

Notes: (a) screening performance without AI, (b) screening performance on general threshold, (c) screening performance on device-specific thresholds. Abbreviations: SDC = Screen-detected breast cancer, IBC = interval breast cancer, AI = Artificial intelligence, CI = confidence interval, CDR = Cancer detection rate

eTable 4: AI Performance on Device-Specific Thresholds with 95% Percentile Bootstrap Confidence Intervals (Stratified Bootstrap Using 10,000 Replications)

	(a)<br>General Threshold	(b)<br>MGD-Specific-Threshold		
	Overall	Siemens Inspiration	Philips<br>L50	GE Pristina
Mammographies<br>- SDC<br>- IBC	22,673<br>115<br>15	11,773<br>62<br>12	5,764<br>33<br>2	5,136<br>20<br>1
Threshold	23<br>(18-37)	37<br>(33-46)	16<br>(5-20)	30<br>(22-61)
Flagged by AI	18.6%<br>(10.0-24.2)	12.9%<br>(8.6-15.7)	8.4%<br>(5.9-34.2)	17.2%<br>(4.2-24.5)
Sensitivity<br>(95%-CI)	83.8%<br>(73.8-91.5)	87.8%<br>(79.7-94.6)	77.1%<br>(71.4-100)	90.5%<br>(76.2-100)
Specificity<br>(95%-CI)	81.8%<br>(76.1-90.4)	87.6%<br>(84.7-91.8)	92.0%<br>(66.1-94.6)	83.1%<br>(75.8-96.2)
Balanced Accuracy (95%-CI)	82.8%<br>(80.1-86.3)	87.7%<br>(84.4-91.7)	84.6%<br>(80.4-91.6)	86.8%<br>(82.2-94.1)
CDR<br>(95%-CI)	4.81<br>(4.23-5.25)	5.52<br>(5.01-5.95)	4.68<br>(4.34-6.07)	3.70<br>(3.12-4.09)

Notes: Computed AI performance metrics based on 10,000 replications in a stratified bootstrap setting. Bootstrap replications were stratified by cancer status, i.e., each replicate contained the same number of cancer and non-cancer cases as the original sample. Threshold selection and performance estimation were repeated within each bootstrap replicate. Confidence intervals are 95% percentile bootstrap intervals from a stratified bootstrap by cancer status. (a) screening performance on computed general threshold, (b) screening performance on computed device-specific thresholds. Abbreviations: SDC = Screen-detected breast cancer, IBC = interval breast cancer, AI = Artificial intelligence, CI = confidence interval, CDR = Cancer detection rate

eTable 5: Current Standard-of-Care (double read with Consensus-Conference) Screening Performance, and AI-Standalone Screening Performance using a General Threshold and Device-Specific Thresholds stratified by Radiologist-assessed Breast Density

	Standard-of-Care Double-Read with Consensus Conferences	AI-Standalone General Threshold	AI-Standalone Device-Specific-Thresholds
Breast Density			
ACR a		1,969 (8.7%)	
ACR b		10,455 (46.1%)	
ACR c		9,650 (42.6%)	
ACR d		599 (2.6%)	
Number of Cases discussed in a Consensus Conference (%)		N/A	N/A
ACR a	82 (4.2%)		
ACR b	593 (5.7%)		
ACR c	686 (7.1%)		
ACR d	64 (10.7%)		
Number of Cases recalled for further investigations (%)		N/A	N/A
ACR a	33 (1.7%)		
ACR b	283 (2.7%)		
ACR c	359 (3.7%)		
ACR d	31 (5.2%)		
Number of AI-Flagged Cases (%)	N/A		
ACR a		267 (13.6%)	159 (8.1%)
ACR b		1958 (18.7%)	1340 (12.8%)
ACR c		1895 (19.6%)	1317 (13.6%)
ACR d		101 (16.9%)	71 (11.9%)
CDR			
ACR a	9 (4.57)	9 (4.57)	9 (4.57)
ACR b	62 (5.93)	55 (5.26)	54 (5.16)
ACR c	39 (4.04)	41 (4.25)	43 (4.46)
ACR d	5 (8.35)	4 (6.68)	5 (8.35)

Notes: Breast density is assessed by the radiologists – either from the consensus conference (if one occurred) or from the second reader. The share of very dense breasts (ACR d) might be low. It is, however, consistent with data from the MSP "donna" from other years and screening sites. Abbreviations: ACR = American College of Radiology, CDR = Cancer detection rate, N/A = Not Applicable
